# Ten-day bismuth-containing quadruple therapy versus 7-day proton pump inhibitor-clarithromycin containing triple therapy as first-line empirical therapy for the *Helicobacter pylori* infection in Korea: a randomized open-label trial

**DOI:** 10.1186/s12876-021-01680-1

**Published:** 2021-03-02

**Authors:** Young-Il Kim, Jong Yeul Lee, Chan Gyoo Kim, Boram Park, Jin Young Park, Il Ju Choi

**Affiliations:** 1 Center for Gastric Cancer, National Cancer Center, 323 Ilsan-ro, Ilsandong-gu, Goyang, Gyeonggi 10408 South Korea; 2 Biostatistics Collaboration Team, Research Core Center, Research Institute, National Cancer Center, Goyang, 10408 Korea; 3 Prevention and Implementation Group, International Agency for Research on Cancer, 69372 Lyon, France

**Keywords:** *Helicobacter pylori*, Quadruple therapy, Triple therapy

## Abstract

**Background:**

This randomized, open-label trial aimed to compare the efficacy of 10-day bismuth-containing quadruple therapy (BQT) with 7-day proton-pump inhibitor-clarithromycin containing standard triple therapy (STT) as an empirical first-line *Helicobacter pylori* therapy.

**Methods:**

Participants with *H. pylori* infection were randomly assigned to either 10-day BQT (daily doses of bismuth 300 mg, four times; lansoprazole 30 mg, twice; metronidazole 500 mg, three times; and tetracycline 500 mg, four times) or 7-day STT (lansoprazole 30 mg; amoxicillin 1,000 mg; and clarithromycin 500 mg; each given twice daily). Participants who failed initial therapy were crossed over to the alternative treatment regimen. Primary outcome was the eradication rates of first-line treatment by intention-to-treat analysis.

**Results:**

Study participants (n = 352) were randomized to receive either 10-day BQT (n = 175) or 7-day STT (n = 177). The BQT-group achieved a significantly higher eradication rate than the STT-group in the intention-to-treat analysis (74.3% vs 57.1%, respectively; *P* = 0.001), modified intention-to-analysis (87.2% [130/149] vs 68.7% [101/147], respectively; *P* < 0.001) and per-protocol analysis (92.9% [105/113] vs 70.1% [94/134], respectively; *P* < 0.001). Although there was no serious adverse event, the compliance was lower with BQT than STT as a higher proportion of participants in the BQT-group discontinued therapy because of adverse events than those in the STT-group (23.1% vs 9.1%, respectively; *P* = 0.001)

**Conclusions:**

Ten-day BQT had higher eradication rates compared to that of the 7-day STT as an empirical first-line treatment for *H. pylori* eradication in Korea.

*Trial registration*: ClinicalTrials.gov, NCT02557932. Registered 23 September 2015, https://clinicaltrials.gov/ct2/show/NCT02557932?term=NCT02557932&draw=2&rank=1.

**Supplementary Information:**

The online version contains supplementary material available at 10.1186/s12876-021-01680-1.

## Background

In the European and North American guidelines on treatment of *Helicobacter pylori*, the proton pump inhibitor-clarithromycin containing standard triple therapy (STT) without antibiotic susceptibility testing is not recommended as the first-line therapy for the eradication of *Helicobacter pylori* in areas of high (> 15%) clarithromycin resistance [1, 2]. In Korea, STT has been recommended as the first-line therapy since the *H. pylori* management guidelines were first reported in 1998 [3–5]. A study using a nationwide online registry of *H. pylori* eradication reported that STT was the most common first-line therapy prescribed (91.8%) in Korea from 2010 to 2015 [6]. However, because of the increased clarithromycin resistance (17.8%-45.9%) [7, 8], two meta-analyses including the studies reported from 1998 to 2013 and a nationwide multicenter randomized study reported in 2019 showed that the eradication rates of STT have decreased to unacceptable levels (63.9–74.6%) in Korea [9–11].

The revised 2013 Korean guideline recommended bismuth-containing quadruple therapy (BQT) as an alternative first-line therapy when clarithromycin resistance is suspected [5]. The European and North American guidelines also recommended the BQT as an empiric first-line therapy in high clarithromycin resistant regions [1, 2]. However, in the nationwide registry database, the prescription rate of BQT as first-line therapy in Korea was low (2.2%) [6]. The low prescription rate of the regimen as first-line therapy is related to the fact that the regimen is reserved for a second-line therapy after the first-line STT failure [5]. The BQT accounts for up to 81% of all prescribed second-line therapies in Korea [6]. It also has a complex dosing schedule and higher risk of adverse events, which might affect compliance with the regimen [12, 13]. Studies that have evaluated the efficacy of BQT in comparison with the STT have been rarely performed in Korea.

Thus, we conducted a randomized trial to investigate whether 10-day BQT is more effective than the 7-day STT as an empirical first-line therapy for *H. pylori* infection.

## Methods

### Study design

This study was a single center, open-label, and randomized trial that was conducted at the National Cancer Center, Korea. The institutional review board at the National Cancer Center approved the trial protocol (approval number: NCC2015-0207), and written informed consent was obtained from all participants before enrollment. The trial was registered with ClinicalTrials.gov (number: NCT02557932).

### Participants

Adult participants (aged ≥ 18 years) were eligible if they had confirmed *H. pylori* infection and one of following conditions: family history of gastric cancer, post-endoscopic resection state for early gastric cancer or adenoma, peptic ulcer disease, or chronic gastritis with non-ulcer dyspepsia. The exclusion criteria included the following: previous *H. pylori* therapy, history of gastrectomy, cancer of another organ in the last 5 years before enrollment, serious concomitant illnesses, contraindication or allergy history of trial medications, and pregnancy.

The *H. pylori* infection status of the participants at enrollment was evaluated with urea breath test, histological examination by Wright-Giemsa staining of biopsy specimens, and/or rapid urease test. Positive *H. pylori* infection status was confirmed as positivity of any of the following conditions: (1) urea breath test, (2) histological test of at least two biopsy sites, or (3) histological test of one biopsy site with rapid urease test.

### Randomization, treatment, and follow-up

Eligible participants were randomly assigned in a 1:1 ratio to receive either BQT for 10 days (lansoprazole 30 mg, twice daily; tripotassium bismuth dicitrate 300 mg, four times daily; tetracycline 500 mg, four times daily; and metronidazole 500 mg, three times daily) or STT for 7 days (lansoprazole 30 mg; clarithromycin 500 mg; and amoxicillin 1,000 mg; all given twice daily). A web-based system using computer-generated permuted random block with a size of 2, 4, or 6 was used for randomization, and the stratification factor was sex. The randomization sequence was strictly concealed from the study investigators.

The success of *H. pylori* therapy was evaluated at least 6 weeks after completion of the therapy, and the urea breath test was used to determine the *H. pylori* status of the participants. Participants who did not achieve *H. pylori* eradication by the initially assigned treatment regimen, received the crossover therapy; 7-day STT for those who failed the 10-day BQT, and 10-day BQT for those who failed the 7-day STT. Another urea breath test was performed at least 6 weeks after completion of the crossover treatment.

### Outcomes

The primary study outcome was the *H. pylori* eradication rate, as assessed by the urea breath test after completion of the first-line therapy in the intention-to-treat (ITT) population. Secondary outcomes included the following: (1) *H. pylori* eradication rate of the first-line therapy in the modified ITT population and per-protocol (PP) population, (2) overall *H. pylori* eradication rate after the crossover therapy in the modified ITT population, and (3) adverse events and compliance. All adverse events were recorded according to the common terminology criteria for adverse events (CTCAE) version 4.0, and the drug compliance was evaluated by counting the returned pills of the study drugs. Serious adverse events were assessed for 30 days after the start of the assigned study therapies. The definitions of adverse event severity and serious adverse events are described in Additional file 1: Supplementrary Table 1 and 2, respectively. Compliance was considered low if the participant had consumed less than 80% of the assigned study drugs.

### Statistical analysis

For sample size calculation, we assumed that the *H. pylori* eradication rates were 74% for the 7-day STT and 86% for the 10-day BQT. The eradication rate of the 7-day STT was based on the following assumptions: (1) the clarithromycin resistance rate was 20%, (2) the eradication rates were 95% for the clarithromycin-susceptible participants and 15% for the clarithromycin-resistant participants, and (3) 7% of the participants of follow-up loss. The eradication rate for the 10-day BQT was based on the assumptions of (1) 7% loss of follow-up participants and (2) 93% eradication rate of the remaining 93% participants based on eradication rates reported by previous studies [14, 15]. With a power of 80% and a two-sided type 1 error of 5%, 175 participants were needed for each treatment arm to detect the 12% difference of eradication rates between two groups (74% vs. 86%). An interim analysis was not planned.

The ITT population included all participants who underwent randomization. Participants who did not perform a follow-up urea breath test were considered as eradication failures in the ITT analysis. We performed the modified ITT analysis as an ad hoc analysis, and of the participants who were included in the ITT population, those who did not start the assigned study drugs and those who did not undergo the follow-up urea breath test were excluded from the modified ITT analysis. The PP population included the participants who took at least 80% of the assigned study drugs and had a follow-up urea breath test result. Eradication rates were assessed with proportion of successful eradication and 95% confidence intervals. The safety analysis was performed for the participants who took at least one dose of the assigned treatment. We used the chi-square test or Fisher’s exact test to analyze the categorical data, and Student’s *t* test to analyze the continuous data. A two-sided *P* value of < 0.05 was considered statistically significant. All statistical analyses were performed using STATA version 16.0 (StataCorp, Texas, USA).

## Results

### Study participants

**Fig. 1:**
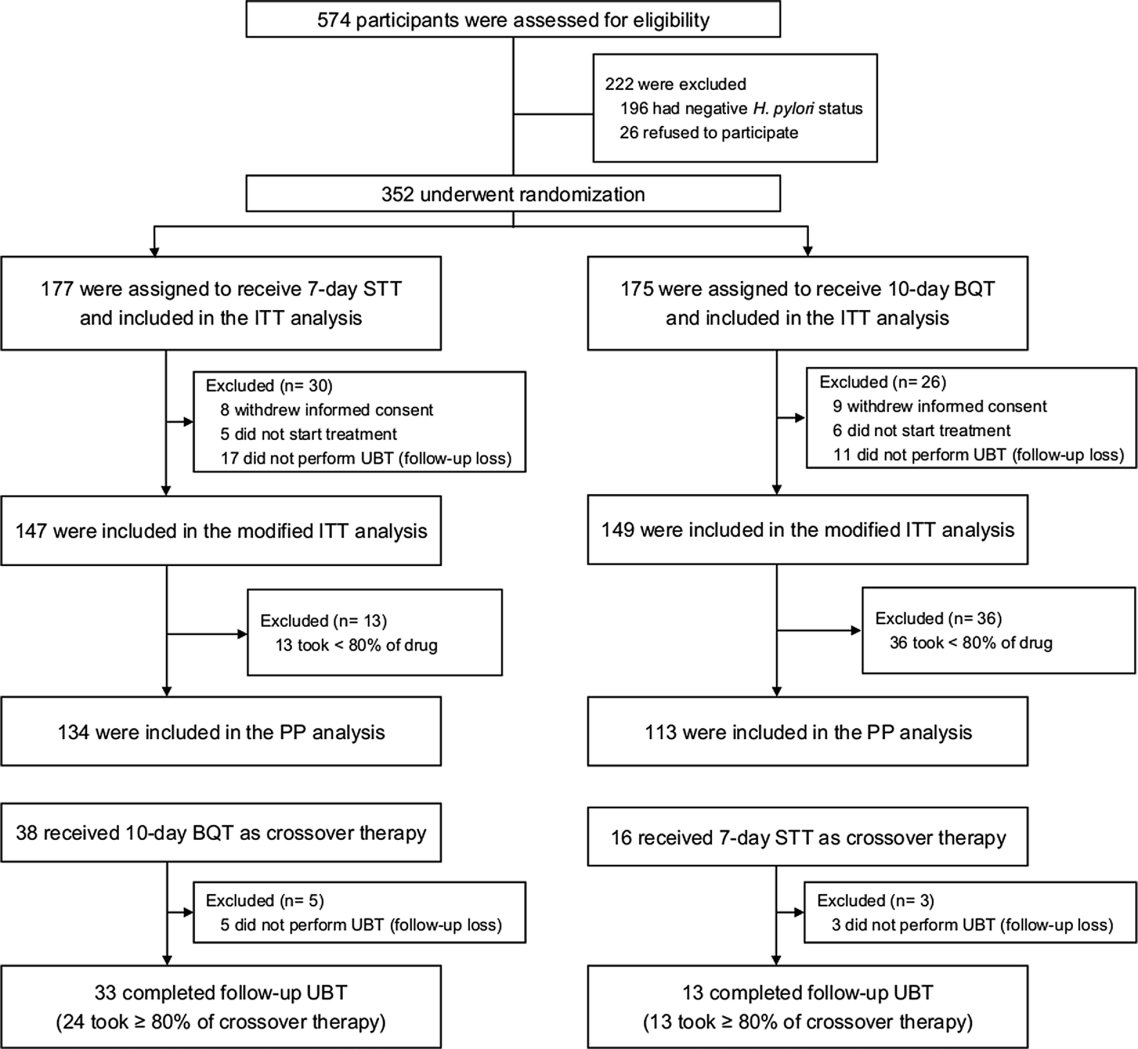
Study flows. STT, proton-pump inhibitor-clarithromycin containing standard triple therapy; BQT, bismuth-containing quadruple therapy; ITT, intention-to-treat; UBT, urea breath test; PP, per-protocol

A total of 574 participants were assessed for eligibility from September 2015 to May 2017. Of these, 352 *H. pylori* positive participants were randomly assigned to receive either 7-day STT (n = 177) or 10-day BQT (n = 175) (Fig. 1). Baseline characteristics were well-balanced between the groups except that the current or former alcohol drinkers were more common in the 10-day BQT group than the 7-day STT group (Table 1).

**Table 1:** Baseline characteristics of all participants

	7-day STT	10-day BQT	*P* value
	(n = 177)	(n = 175)	
Age (year), Mean ± SD	55.6 ± 11.3	53.9 ± 10.7	0.147
Sex, no. (%)			0.920
Male	83 (46.9)	83 (47.4)	
Female	94 (53.1)	92 (52.6)	
Smoking, no. (%)			0.196
Never	116 (65.5)	103 (58.9)	
Current or former smoker	61 (34.5)	72 (41.1)	
Alcohol, no. (%)			0.028
Never	82 (46.3)	61 (34.9)	
Current or former alcohol drinker	95 (53.7)	114 (65.1)	
Family history of gastric cancer, no. (%)	65 (36.7)	61 (34.9)	0.715
Coexisting illness, no. (%)			
Hypertension	37 (20.9)	43 (24.6)	0.412
Diabetes mellitus	11 (6.2)	13 (7.4)	0.651
Indications for *H. pylori* treatment, no. (%)			
Post-ESD for adenoma or gastric cancer	55 (31.1)	51 (29.1)	0.693
Peptic ulcer disease	22 (12.4)	27 (15.4)	0.416
Chronic gastritis with non-ulcer dyspepsia	100 (56.5)	97 (55.4)	0.840

STT, proton-pump inhibitor-clarithromycin containing standard triple therapy; BQT, bismuth-containing quadruple therapy; SD, standard deviation; ESD, endoscopic submucosal dissection

### Eradication rates of first-line therapy

In the ITT analysis, the eradication rate with the first-line therapy was significantly higher in the participants treated with the 10-day BQT (74.3%, 130/175 participants) than in those treated with the 7-day STT (57.1%, 101/177 participants; *P* = 0.001) (Table 2). The post hoc modified ITT analysis also showed significantly higher rate of eradication in the BQT group (87.2%, 130/149 participants) than in the STT group (68.7%, 101/147 participants; *P* < 0.001). In the PP analysis, the BQT group (92.9%, 105/113 participants) had a higher eradication rate compared with the STT group (70.1%, 94/134 participants; *P* < 0.001).

**Table 2:** First-line, crossover and overall eradication rates

	7-day STT		10-day BQT		*P* value
	no./total no. (%)	95% CI	no./total no. (%)	95% CI	
Eradication rate with first-line therapy					
Intention-to-treat analysis^a^	101/177 (57.1)	49.4–64.5	130/175 (74.3)	67.1–80.6	0.001
Modified intention-to-treat analysis^b^	101/147 (68.7)	60.5–76.1	130/149 (87.2)	80.8–92.1	< 0.001
Per-protocol analysis^c^	94/134 (70.1)	61.6–77.7	105/113 (92.9)	86.5–96.9	< 0.001
Eradication rate with crossover therapy					
Modified intention-to-treat analysis^b^	30/33 (90.9)	75.7–98.1	11/13 (84.6)	54.6–98.1	0.612
Per-protocol analysis^c^	24/24 (100)	85.8–100	11/13 (84.6)	54.6–98.1	0.117
Overall eradication rate after crossover therapy					
Intention-to-treat analysis^a^	131/177 (74.0)	66.9–80.3	141/175 (80.6)	73.9–86.2	0.142
Modified intention-to-treat analysis^b^	131/134 (97.8)	93.6–99.5	141/143 (98.6)	95.0–99.8	0.676
Per-protocol analysis^c^	117/117 (100)	96.9–100	108/109 (99.1)	95.0–100.0	0.482

STT, proton-pump inhibitor-clarithromycin containing standard triple therapy; BQT, bismuth-containing quadruple therapy; CI, confidence interval

^a^This analysis was performed in the intention-to-treat population, which included all participants who underwent randomization

^b^The modified intention-to-treat analysis for the first-line therapy and crossover therapy was performed in participants who started the assigned therapies and underwent the follow-up urea breath test. For overall eradication rate after crossover therapy, participants who achieved eradication success with first-line therapy and those who started the crossover therapy after failure of first-line therapy and underwent the follow-up urea breath test were included

^c^The per-protocol analysis for the first-line therapy and crossover therapy was performed in participants who took at least 80% of the therapies and underwent the follow-up urea breath test. For overall eradication rate after crossover therapy, participants who achieved eradication success with first-line therapy in the per-protocol population and those who took at least 80% of the crossover therapy after failure of first-line therapy and underwent the follow-up urea breath test were included

### Overall eradication rates after the crossover therapy

In the modified ITT population for the overall eradication rate, 134 participants from the STT group and 143 from the BQT group were included. In the 7-day STT group, 101 participants who achieved success of *H. pylori* eradication with the first-line and 33 participants who started the crossover therapy and underwent follow-up with the urea breath test after the therapy were included. In the 10-day BQT group, 130 *H. pylori*-eradicated participants with the first-line therapy and 13 started the crossover therapy were included. The eradication rate of the 10-day BQT as a crossover therapy was 90.9% (30/33 participants) and that of the 7-day STT was 84.6% (11/13 participants). Overall eradication rates were not significantly different between the groups (97.8% [131/134 participants] for the STT group and 98.6% [141/143 participants] for the BQT group; *P* = 0.676; Table 2). In the ITT and PP analyses, the overall eradication rates were also not different between the groups.

### Adverse events and compliance

In the safety population for the first-line therapy, 164 participants in the 7-day STT and 160 in the 10-day BQT who underwent randomization and received at least one dose allocated treatment were included. The proportion of any adverse events was not significantly different between the treatment groups (57.3% in the STT group vs. 67.5% in the BQT group; *P* = 0.059) (Table 3). However, the incidence of nausea, vomiting, headache, dyspepsia, general weakness, and anorexia was significantly higher in participants treated with the BQT. Meanwhile, the STT group had a higher proportion of participants with an alteration in taste. All adverse events were grade 1 (mild) or grade 2 (moderate) (Additional file 1: Supplementary Table 3), and none of the participants had serious adverse event. Data on the adverse events related to crossover treatments are shown in Additional file 1: Supplementary Table 4.

**Table 3:** Adverse events and compliance^a^

Events	7-day STT	10-day BQT	*P* value
	(n = 164)	(n = 160)	
Adverse events, no. (%)			
Taste alteration	47 (28.7)	7 (4.4)	< 0.001
Nausea	14 (8.5)	38 (23.8)	< 0.001
Vomiting	0 (0)	10 (6.3)	0.001
Diarrhea	25 (15.2)	28 (17.5)	0.583
Abdominal discomfort	25 (15.2)	35 (21.9)	0.124
Skin rash or urticaria	2 (1.2)	6 (3.8)	0.170
Dizziness	9 (5.5)	15 (9.4)	0.182
Headache	5 (3.0)	19 (11.9)	0.002
Insomnia	5 (3.0)	2 (1.3)	0.448
Dyspepsia	17 (10.4)	33 (20.6)	0.011
General weakness	3 (1.8)	13 (8.1)	0.009
Dyspnea	1 (0.6)	1 (0.6)	> 0.999
Anorexia	0 (0)	9 (5.6)	0.002
Dry mouth	5 (3.0)	2 (1.3)	0.448
Chest discomfort	0 (0)	2 (1.3)	0.243
Any adverse event,^b^ no. (%)	94 (57.3)	108 (67.5)	0.059
Compliance with the study drugs, no. (%)			< 0.001
Participants who took ≥ 80% of study drugs	145 (88.4)	117 (73.1)	
Participants who took < 80% of study drugs	19 (11.6)	43 (26.9)	
Participants with low compliance due to adverse events,^c^ no. (%)	15 (9.1)	37 (23.1)	0.001

STT, proton-pump inhibitor-clarithromycin containing standard triple therapy; BQT, bismuth-containing quadruple therapy

^a^This analysis was performed in the safety population, which included all participants who underwent randomization and received at least one dose allocated treatment. Thirteen participants in the 7-day PPI-clarithromycin containing triple therapy group and fifteen in the 10-day bismuth-containing quadruple therapy group were excluded in this analysis

^b^There was no participant who had any serious adverse events including death, life-threatening events, hospitalization, prolongation of existing hospitalization, persistent or significant disability/incapacity, congenital anomaly/birth defect, and medically important event or reaction

^c^Low compliance was defined when participant took less than 80% of the study drugs

Compliance with the study drugs was worse in participants treated with the BQT than in those treated with the STT. The proportion of the participants with low compliance, who took less than 80% of the assigned study drugs, was 26.9% (43/160 participants) in those treated with the BQT and 11.6% (19/164 participants) in participants treated with the STT (*P* < 0.001; Table 3). The BQT group had a higher proportion of participants who discontinued the assigned study drugs and consumed less than 80% of the assigned study drugs because of the adverse events than in the STT group (23.1% vs. 9.1%, respectively; *P* = 0.001).

### Eradication rates with the first-line therapy according to drug compliance

**Fig. 2:**
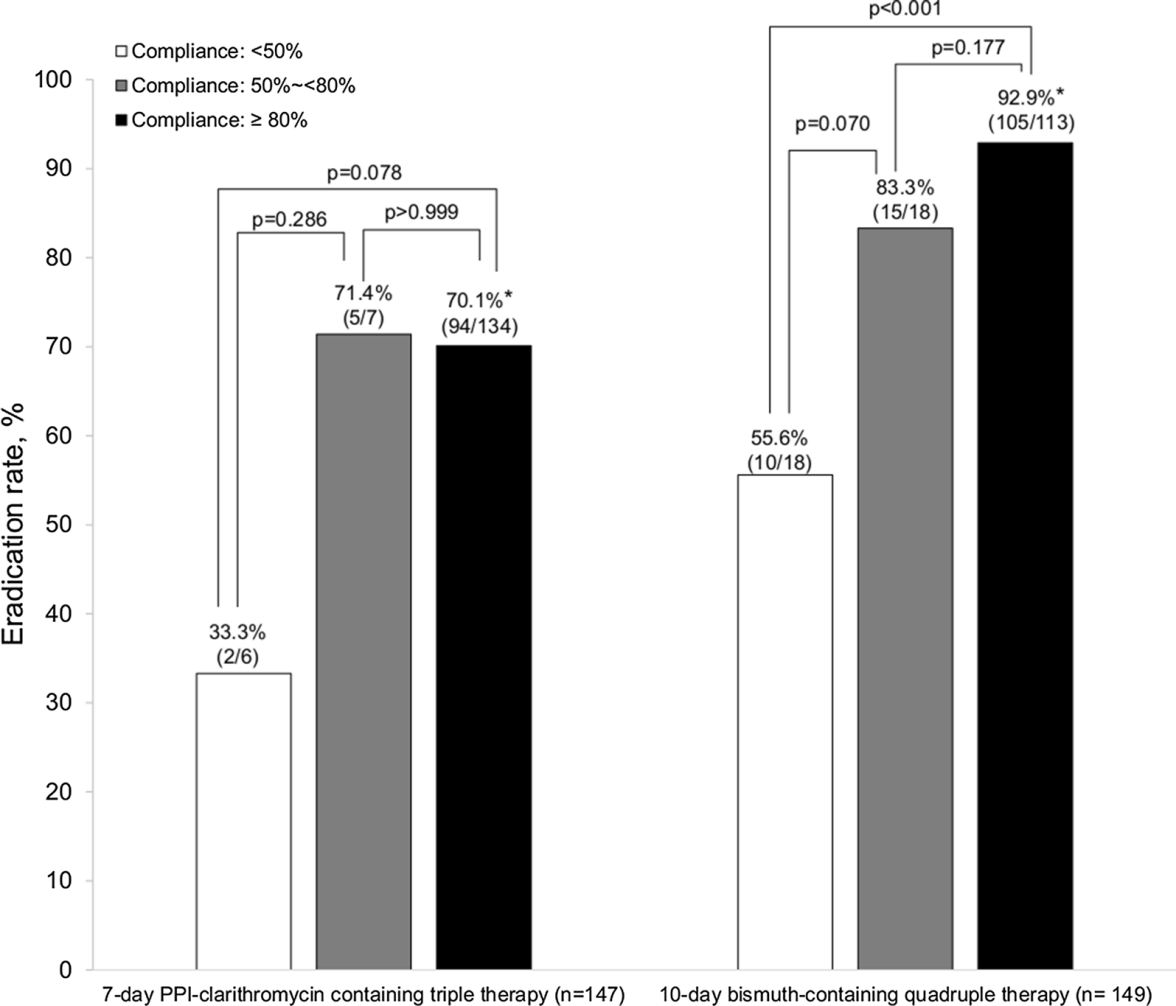
Eradication rates of first-line therapy according to study drug compliance (*P value < 0.001 between 7-day PPI-clarithromycin containing triple therapy and 10-day bismuth-containing quadruple therapy). PPI, proton-pump inhibitor

Eradication rates after the initial first-line therapy according to the study drug compliance are presented in Additional file 1: Supplementary Table 5. In the BQT group, the eradication rate of participants who consumed ≥ 80% of the assigned study drugs (92.9%, 105/113 participants) was significantly higher than that of participants who consumed < 50% of the drugs (55.6%, 10/18 participants; *P* < 0.001) (Fig. 2). However, despite low compliance (< 80%) to the study drugs, the eradication rate was 83.3% (15/18 participants) when participants consumed at least 50% of the drugs. No significant difference was found in the eradication rate between participants who consumed ≥ 80% and 50–80% of the assigned study drugs (*P* = 0.177).

The eradication rate of the participants who completed 50–80% of the STT (71.4%, 5/7 participants) was not different compared with those who completed ≥ 80% of that therapy (70.1%, 94/134 participants; *P* > 0.999) (Fig. 2). However, a low eradication rate (33.3%, 2/6 participants) was reported in those who completed less than 50% of that therapy, although there were no significant differences in eradication rates compared with those who completed ≥ 80% or 50%-80% of the therapy.

## Discussion

This randomized trial has shown that the 10-day BQT is superior to the 7-day STT as an empirical first-line therapy for the *H. pylori* infection. Although the eradication rate of first-line BQT therapy was low in the ITT population (74.3%) because of the poor compliance, that in the PP population was good (92.9%). Overall combined eradication rates after the crossover therapy were similarly high in both therapy groups.

Our data showed that the eradication rates of the 7-day STT were 57% in the ITT analysis, 69% in the modified ITT analysis and 70% in the PP analysis. The low eradication rates are similar to those reported in a nationwide randomized trial conducted in Korea between 2016 and 2018, which reported the eradication rates of 7-day STT to be 63.9% in the ITT analysis and 71.4% in the PP analysis [11]. According to a meta-analysis of nine randomized controlled trials between 2005 and 2013, the overall eradication rate of the STT was 68.5% in the ITT analysis, and it was inferior to other regimens including the sequential therapy or concomitant therapy as a first-line therapy [9]. In another meta-analysis including 38 randomized controlled trials and 66 observational studies conducted between 1998 and 2013, it was reported that the overall eradication rate of first-line STT was 74.6% in the ITT analysis, and the rate showed a significantly decreasing tendency from 1998 to 2013 in Korea [10]. The decreasing eradication rates were mainly because of high occurrence of clarithromycin resistance (> 15%) in Korea [7, 8, 16]. The acceptable targets for the eradication rates of *H. pylori* therapy regimens have been suggested to be ≥ 85% by the ITT analysis and ≥ 90% by the PP analysis [17]. Considering these suggestions, the eradication rates of STT reported in our trial and previous studies indicate that STT is unacceptable as a first-line therapy.

In our trial, the crossover 7-day STT in participants who failed to achieve successful eradication in the initial 10-day BQT group showed a higher eradication rate (84.6%) compared with the first-line 7-day STT (68.7%). The better eradication rate might be associated with a high compliance with the crossover 7-day STT (all participants took ≥ 80% of provided drugs). In addition, there might be a possibility that most of the 13 participants who received the crossover 7-day STT had clarithromycin-susceptible organism, although clarithromycin resistance was not evaluated.

Our trial showed that the eradication rate of 10-day BQT was 74% in the ITT analysis, 87% in the modified ITT analysis and 93% in the PP analysis, and it was significantly higher than that of the 7-day STT as an empirical first-line therapy. The high eradication rates in our trial were consistent with those reported in previous studies investigating the first-line 10-day BQT in comparison to that of the other regimens (80–93% in the intention-to-treat analysis and 93–99% in the per-protocol analysis) [14, 15, 18]. Thus, the 10-day BQT can be an effective empirical first-line therapy alternative to the 7-day STT in Korea.

However, eradication rate of the BQT was 74% in the ITT analysis, which is unacceptable according to the suggested acceptable target eradication rates of ≥ 85% by the ITT analysis for the *H. pylori* therapy [17]. In our study, the rate of participants (15.9%, 56/352 participants) who were lost to follow-up or declined to participate was higher than that we expected (7%) at sample size calculation. Of 56 participants, 43 (76.8%) were enrolled for the treatment of *H. pylori* infection due to chronic gastritis with non-ulcer dyspepsia. Among indications for *H. pylori* treatment of participants who declined to participate in the study or were lost to follow-up, the proportion of chronic gastritis with non-ulcer dyspepsia (21.8%, 43/197) was a higher than that of post-ESD status (6.6%, 7/106) and peptic ulcer disease (12.2%, 6/49) (Additional file 1: Supplementary Table 6). Because chronic gastritis is not an indication for *H. pylori* eradication in Korea [5], the participants with chronic gastritis might have changed their mind after informed consent and refused to continue participating in the clinical trial. In contrast, the proportion of participants who withdrew consent or lost to follow-up immediately after study enrollment and consequently excluded from the ITT analysis was low in studies conducted outside Korea [14, 15, 18]. Therefore, in our trial, the modified ITT analysis seems to be more reasonable than the ITT analysis in comparing the efficacy with the trials performed in other countries.

Drug compliance with the BQT is important to achieve successful treatment of *H. pylori*. Complex dosing schedule and high frequency of adverse events could reduce compliance for the BQT [12, 13]. Regarding the complexity of dosing schedule, previous studies have reported that the modification of drug schedule (twice a day schedule) or use of a single drug containing bismuth, metronidazole, and tetracycline was effective and showed a good compliance [16, 19, 20]. In the present study, low compliance with the BQT seemed to be mainly because of the adverse events, although the frequency of any adverse events was not significantly different between the groups as it was compensated by a higher frequency of taste disturbance observed in the STT group. Most participants treated with the STT could tolerate the adverse events and continued the medication. In a meta-analysis of 41 studies reporting adverse events of the STT, the frequency of adverse events was 20.4%, but the proportion of participants who stopped therapy because of adverse events was low at 1.8% [10]. Meanwhile, in participants treated with the BQT in the current study, adverse events that affected the drug compliance including nausea, vomiting, headache, dyspepsia, general weakness, and anorexia, were more frequent. Although there was no serious adverse event, BQT group had a higher proportion of participants (23.1%) discontinued and took less than 80% of the assigned study drugs because of the adverse events. The eradication rate of the BQT in participants who took less than 80% of the assigned study drugs (83% in those who consumed 50% to 80% of the drugs, and 56% in those who consumed less than 50% of the drugs) did not reach the acceptable eradication rate of ≥ 85% [17]. Thus, it is important to provide detailed information to participants on potential adverse events that can occur and emphasize that they should take the prescribed drugs as much as possible if the adverse events are tolerable, to achieve high eradication rate with the BQT. To improve the compliance and reduce adverse events in the 10-day BQT group, additional efforts are needed as follows: (1) use of a single three-in-one capsule containing bismuth, metronidazole, and tetracycline to increase patient convenience [14], (2) modification of drug administration schedule or dosing [13], or (3) adding probiotics [21].

The resistance rates of clarithromycin or metronidazole have been reported to be high (17.8–45.9% and 29.5–63.2%, respectively) in Korea [7, 8, 22]. A randomized trial from Taiwan reported that the 10-day BQT was effective as first-line and second-line therapy, with a success rate of > 90%, regardless of the resistance to clarithromycin or metronidazole [18]. Similar to that study results, the BQT in our study was effective and showed a high eradication rate of 91% as a crossover therapy after failure of the initial STT. Participants who were initially treated with the 7-day STT could achieve an overall eradication rate of 98% after the crossover therapies, which is the current practice as recommended in the Korean guideline [5]. For those treated by the initial 10-day BQT, overall eradication rate after crossover therapy was also high at 99%. Thus, empirical *H. pylori* therapy consisting of those two regimens could provide final high eradication rates without antibiotic susceptibility testing after the crossover treatment.

In our trial, treatment duration was not the same for the two therapy groups (7 days for STT and 10 days for BQT). International guidelines recommend 14 days for the STT in areas of low clarithromycin resistance [2, 23], and 10 or 14 days for the BQT [1, 2, 23–25]. In Korea, 7 to 14 days have been recommended for both regimens [5]. Despite a controversy regarding the optimal duration of STT, meta-analyses reported that extending the duration of the regimen from 7 to 10 or 14 days did not increase the eradication rates significantly in Korea [9, 10]. In addition, 7 days duration for STT is the most commonly prescribed regimen in Korea (84% of all prescribed first-line regimens) [6]. For the BQT, the eradication rates were higher in the 14 days treatment period as compared to that of the 7 days regimen [26, 27], whereas there was no significant difference between the 14 days and 10 days regimen [25]. Thus, considering the guidelines, drug compliance, and actual clinical practices in Korea, we selected 7 days for the STT and 10 days for the BQT for comparison in the current study.

As far as we know, this study is the first well-designed prospective randomized controlled trial comparing the 10-day BQT to the 7-day STT as the empirical first-line therapy in Korea. However, there are several limitations to this study. First, we did not evaluate the antibiotic resistance as this study was planned to evaluate the eradication rates in the empirical treatment setting. Second, the proportion of participants who did not return to follow-up visit, including withdrawal from study participation and refusal to receive the assigned study drug, was higher than expected. Thus, we also performed the ad hoc modified ITT analysis after excluding those participants.

## Conclusions

Our trial provides evidence that the 10-day BQT is highly effective in eradicating *H. pylori* as an empirical first-line therapy in Korea. Because the first-line 7-day STT has an unacceptable efficacy, the current Korean guidelines for *H. pylori* management need to be revised to include BQT as the first-line therapy.

## Supplementary Information

**Additional file 1:** Supplementary materials.

## Data Availability

The datasets used and analyzed in the current study are available from the corresponding author on reasonable request.

## Abbreviations

STT: Proton-pump inhibitor-clarithromycin containing standard triple therapy
BQT: Bismuth-containing quadruple therapy
ITT: Intention-to-treat
PP: Per-protocol
CTCAE: Common terminology criteria for adverse event
