# Supplementary material for: Ten-day bismuth-containing quadruple therapy versus 7-day proton pump inhibitor-clarithromycin containing triple therapy as first-line empirical therapy for the Helicobacter pylori infection in Korea: a randomized open-label trial
Source: BMC Gastroenterol. 2021 Mar 2;21:95. doi: 10.1186/s12876-021-01680-1 (PMC7923489; doi:10.1186/s12876-021-01680-1)
Supplement: Supplementary file 1 — Additional file 1: Supplementary materials. [file 12876_2021_1680_MOESM1_ESM.docx]

**Supplementary materials**

**Ten-day bismuth-containing quadruple therapy versus 7-day proton pump inhibitor-clarithromycin containing triple therapy as first-line empirical therapy for the *Helicobacter pylori* infection in Korea: A randomized open-label trial**

Supplementary table 1. Definition of severity for adverse events

| Severity of adverse event**^a^** | |
| --- | --- |
| Grade 1 | **Mild**; asymptomatic or mild symptoms; clinical or diagnostic observations only; intervention not indicated. |
| Grade 2 | **Moderate**; minimal, local or noninvasive intervention indicated; limiting age-appropriate instrumental activities of daily living. (Instrumental activities of daily living refer to preparing meals, shopping for groceries or clothes, using the telephone, managing money, etc.) |
| Grade 3 | **Severe or medically significant but not immediately life-threatening**; hospitalization or prolongation of hospitalization indicated; disabling; limiting self care activities of daily living. (Self care activities of daily living refer to bathing, dressing and undressing, feeding self, using the toilet, taking medications, and not bedridden.) |
| Grade 4 | **Life-threatening consequences**; urgent intervention indicated. |
| Grade 5 | **Death related to adverse event**. |
| **^a^**The definitions of severity grades for adverse events are based on the general guideline of the common terminology criteria for adverse events (CTCAE) version 4.0. | |

Supplementary table 2. Definition of serious adverse events in the present trial

| Serious adverse events related to the study drugs were defined as following conditions^a^  Death  Life-threatening condition  Hospitalization  Prolongation of existing hospitalization  Persistent or significant disability or incapacity  Congenital anomaly or birth defect  Medically important event or reactions |
| --- |
| ^a^Serious adverse events were assessed for 30 days after the start of study drugs. |

Supplementary table 3. Severity of adverse events associated with first-line treatment**^a^**

|  | 7-day STT | | |  | 10-day BQT | | |
| --- | --- | --- | --- | --- | --- | --- | --- |
|  | (n=164) | | |  | (n=160) | | |
|  | Total | Grade 1 | Grade 2 |  | Total | Grade 1 | Grade 2 |
| Adverse events, no. (%) |  |  |  |  |  |  |  |
| Taste alteration | 47 (28.7) | 45 (27.4) | 2 (1.2) |  | 7 (4.4) | 7 (4.4) | 0 (0) |
| Nausea | 14 (8.5) | 13 (7.9) | 1 (0.6) |  | 38 (23.8) | 31 (19.4) | 7 (4.4) |
| Vomiting | 0 (0) | 0 (0) | 0 (0) |  | 10 (6.3) | 10 (6.3) | 0 (0) |
| Diarrhea | 25 (15.2) | 22 (13.4) | 3 (1.8) |  | 28 (17.5) | 26 (16.3) | 2 (1.3) |
| Abdominal discomfort | 25 (15.2) | 19 (11.6) | 6 (3.7) |  | 35 (21.9) | 31 (19.4) | 4 (2.5) |
| Skin rash or urticaria | 2 (1.2) | 2 (1.2) | 0 (0) |  | 6 (3.8) | 4 (2.5) | 2 (1.3) |
| Dizziness | 9 (5.5) | 9 (5.5) | 0 (0) |  | 15 (9.4) | 13 (8.1) | 2 (1.3) |
| Headache | 5 (3.0) | 4 (2.4) | 1 (0.6) |  | 19 (11.9) | 17 (10.6) | 2 (1.3) |
| Insomnia | 5 (3.0) | 4 (2.4) | 1 (0.6) |  | 2 (1.3) | 2 (1.3) | 0 (0) |
| Dyspepsia | 17 (10.4) | 14 (8.5) | 3 (1.8) |  | 33 (20.6) | 23 (14.4) | 10 (6.3) |
| General weakness | 3 (1.8) | 3 (1.8) | 0 (0) |  | 13 (8.1) | 10 (6.3) | 3 (1.9) |
| Dyspnea | 1 (0.6) | 1 (0.6) | 0 (0) |  | 1 (0.6) | 1 (0.6) | 0 (0) |
| Anorexia | 0 (0) | 0 (0) | 0 (0) |  | 9 (5.6) | 7 (4.4) | 2 (1.3) |
| Dry mouth | 5 (3.0) | 5 (3.0) | 0 (0) |  | 2 (1.3) | 2 (1.3) | 0 (0) |
| Chest discomfort | 0 (0) | 0 (0) | 0 (0) |  | 2 (1.3) | 1 (0.6) | 1 (0.6) |
| STT, proton-pump inhibitor-clarithromycin containing standard triple therapy; BQT, bismuth-containing quadruple therapy. | | | | | | | |
| **^a^**This analysis was performed in the safety population, which included all participants who underwent randomization and received at least one dose allocated treatment. Thirteen participants in the 7-day PPI-clarithromycin containing triple therapy group and fifteen in the 10-day bismuth-containing quadruple therapy group were excluded in this analysis. Grades for adverse events were recorded according to the definitions of the common terminology criteria for adverse events (CTCAE) version 4.0. | | | | | | | |

Supplementary table 4. Adverse events and compliance to cross-over treatment**^a^**

| Events | 7-day STT | 10-day BQT | *P* value |
| --- | --- | --- | --- |
|  | (n=14) | (n=36) |  |
| Adverse events, no. (%) |  |  |  |
| Taste alteration | 5 (35.7) | 2 (5.6) | 0.014 |
| Nausea | 0 (0) | 10 (27.8) | 0.045 |
| Vomiting | 0 (0) | 2 (5.6) | >0.999 |
| Diarrhea | 6 (42.9) | 5 (13.9) | 0.052 |
| Abdominal discomfort | 2 (14.3) | 5 (13.9) | >0.999 |
| Skin rash or urticaria | 0 (0) | 1 (2.8) | >0.999 |
| Dizziness | 1 (7.1) | 1 (2.8) | 0.486 |
| Headache | 0 (0) | 4 (11.1) | 0.566 |
| Dyspepsia | 0 (0) | 8 (22.2) | 0.087 |
| General weakness | 0 (0) | 3 (8.3) | 0.55 |
| Dyspnea | 0 (0) | 1 (2.8) | > 0.99 |
| Anorexia | 0 (0) | 1 (2.8) | > 0.99 |
| Any adverse event,^b^ no. (%) | 7 (50.0) | 24 (66.7) | 0.276 |
| Participant who took at least 80% of the Study medications, no. (%) | 13 (92.9) | 24 (66.7) | 0.078 |
| Proton-pump inhibitor-clarithromycin containing standard triple therapy, STT; Bismuth-containing quadruple therapy, BQT.  **^a^**In this analysis, all participants who received at least one dose cross-over treatment were included. Two participants who received bismuth-containing quadruple therapy and two participants who received 7-day PPI-clarithromycin containing triple therapy were excluded in this analysis, because of follow-up loss. | | | |
| **^b^**There was no participant who had any serious adverse events. | | | |

Supplementary table 5. Eradication rates with the first-line therapy according to drug compliance

| Eradication success, %  (no./total no.) | 7-day STT | 10-day BQT |
| --- | --- | --- |
|  | (n=147) | (n=149) |
| Compliance |  |  |
| < 10% | 0 (0/1) | 66.7 (2/3) |
| 10% - < 20% | 0 (0/1) | 0 (0/2) |
| 20% - < 30% | 0 (0/2) | 66.7 (2/3) |
| 30% - < 40% | - | 75.0 (6/8) |
| 40% - < 50% | 100 (2/2) | 0 (0/2) |
| 50% - < 60% | 50.0 (1/2) | 87.5 (7/8) |
| 60% - < 70% | - | 100 (2/2) |
| 70% - < 80% | 80.0 (4/5) | 75.0 (6/8) |
| 80% - < 90% | 100 (2/2) | 75.0 (3/4) |
| 90% - 100% | 69.7 (92/132) | 93.6 (102/109) |
| Total | 68.7 (101/147) | 87.2 (130/149) |
| Proton-pump inhibitor-clarithromycin containing standard triple therapy, STT; Bismuth-containing quadruple therapy, BQT. | | |

Supplementary table 6. Proportion of participants who decline to participate in the study or were lost to follow-up after randomization according to indications for *H. pylori* treatment

|  | Total | 7-day STT | 10-day BQT |
| --- | --- | --- | --- |
|  | (n=352) | (n=177) | (n=175) |
| Indications for *H. pylori* treatment, no. (%) |  |  |  |
| Post-ESD for adenoma or gastric cancer | 7/106 (6.6) | 6/55 (10.9) | 1/51 (2.0) |
| Peptic ulcer disease | 6/49 (12.2) | 2/22 (9.1) | 4/27 (14.8) |
| Chronic gastritis with non-ulcer dyspepsia | 43/197 (21.8) | 22/100 (22.0) | 21/97 (21.6) |
| *P* value | 0.001 | 0.150 | 0.002 |
| STT, proton-pump inhibitor-clarithromycin containing standard triple therapy; BQT, bismuth-containing quadruple therapy | | | |
